# Os-Artificial Impugned

**Published:** 1869-12

**Authors:** 


### "OS-ARTIFICIAL IMPUGNED."

My critic, writing under the above head line in the November REGISTER, has produced a review which, in some of its parts, is quite too erudite for my pen. When I had read to the end of his second paragraph, the first time, I am able now to remember that I seemed to be released from all sublunary things. How long I floated a thousand feet or more in the regions of space that surround terra-firma, I can not now tell. All I can say is, that there came a time when I realized that I was not yet done with materiality, and with this working consciousness I perceived that I might still use my privilege, perhaps duty, of dealing with things on my immediate plane, and thus I resolved, that, though I could not grapple with the erudition of W. F. M., I could say in my own way something further of os-artificial, because the matter concerned Dentists and Dentistry.

"*To me there is nothing reasonable in the idea that oxy-chloride of zinc can induce the re-formation of natural bone over exposed pulps.*" Upon this matter I am not only not changed, but every month growing more incredulous. I think it just barely probable that exposed pulps may be bridged over where the recuperative forces are strong and the pulp is kept covered a sufficient length of time, but unquestionably this process will go on quite as well under one non-conducting substance that will remain in *situ* and impervious to fluids, as another. This is what I claim and what my experience will not permit me to doubt. "*It is a foreign substance and an irritant.*" Does W. F. M. think it is not? And is the "*styptic colloid*" covering less a foreign substance?

But my critic is confident that the "*positive failures are more in the Dentist than in the agent.*" Is the relation of arseneous acid to mucous membrane changed, or its action thereon modified by the skill, or otherwise, of the hand that causes them to come in contact? And how does W. F. M. know that the larger number are "*careless and heedless,* and unpliedly unskillful in their manipulations with the agent in question. I can excuse the egotism which prompts such assumption, for it is a quality of the mind and is no more to be quarreled with than pug noses or red hair. And the confidence of the reviewer sustained by his self reliance, makes him clearly ask to be understood as saying that *he* is not one of, the "*careless and heedless,*" and unskillful.

He means to educate the miscellaneous contributor, and "*clear him up a little.*" His lessons will be worth more ten years hence than now, if he should have, during that time, taken careful note of a great many cases thus treated, and then render a tabular statement of the per centages of successes and failures. And in that way he perhaps also can contribute something to Dental literature that will be worth filing. I do not now pretend to say what the results of such observations are to be, but I think it quite probable that my friend's confidence in the reliability of the oxychloride as a "*pulp preserver*" would be materially toned down. We have no guides beside our experiences and those of others working with us, and of these our own is first, others are aids. We shall see, let us wait.

And again. "Its popularity is steadily gaining." With some it is; with others loosing. It has been my experience, to have witnessed many cherished births of conceived right ways of doing things in Dental practice that have steadily, for a time, ascended towards the zenith, and then declined and set in endless night. I do not say it will be so with the os-artificial as a pulp preserver, but I am for patient waiting after careful trial.

I do not know why it should have been said that I impugned the os-artificial. I wished to say that perhaps too much is being claimed for it in advance of sufficient trial, and I gave my reasons for so thinking. But then I told him I used it and what for. No man would rejoice over its success more sincerely than myself, and I hope that I shall not be outdone in gratitude to the benefactors of our profession who bring real discoveries to us. In the meantime we must all keep very much to our own experiences.

I am glad to see this agent thoroughly tested by earnest men, and do not intend to raise uncalled for issues before the time, and therefore will not take further time or space at present in writing down in extenso all my reason for the want of confidence I have expressed. I will not hesitate to do so, however, at more length should the occasion arise.

And now, if readers will indulgently excuse the repetitions of the personal pronoun, first person, which has seemed unavoidable, I will for the present dismiss the theme by saying that I never expect to know that the oxychloride of zinc can induce the growth of natural bone when placed over the exposed membrane of nerve pulp. The nerve membrane is an organized, vital and exceedingly sensative tissue that will not long tolerate the proximity of any inorganic, and therefore lifeless matter, and I have no doubt whatever, that under the treatment proposed, even by the best skill, by far the greatest number of pulps will at least be found to have perished. MISCELLANIES.

